# Retrieval-augmented generation salvages poor performance from large language models in answering microbiology-specific multiple-choice questions

**DOI:** 10.1128/jcm.01624-24

**Published:** 2025-02-11

**Authors:** Grace Perkins, Neil W. Anderson, Nicholas C. Spies

**Affiliations:** 1 Department of Pathology and Immunology, Washington University School of Medicine 12275 , St. Louis, Missouri, USA; 2 Department of Pathology, University Hospitals Cleveland Medical Center 114516 , Cleveland, Ohio, USA; 3 Department of Pathology, University of Utah 161530 , Salt Lake City, Utah, USA; 4 Division of Research and Innovation, ARUP Laboratories 33294 , Salt Lake City, Utah, USA; Boston Children's Hospital, Boston, Massachusetts, USA

**Keywords:** artificial intelligence, medical education, large language models

## LETTER

Large language models (LLMs) have recently demonstrated remarkable proficiency in various natural language processing tasks within healthcare, including medical question-answering (1, 2). The opportunity for increased efficiency within the clinical laboratory makes these tools a promising area of active research. However, LLMs are not without limitations. Perhaps most notable of these is their tendency to “hallucinate”—or generate a response that appears plausible at first glance but contains factual inaccuracies. Hallucinations by generative artificial intelligence (AI) models in the field of clinical microbiology may lead to erroneous organism identification, incorrect antibiotic resistance profiles, and ultimately to inappropriate treatment and potential patient harm.

In this study, we sought to evaluate the performance of two current LLMs, GPT-4 (OpenAI, San Francisco, CA, USA) and Claude3 Sonnet (Anthropic, San Francisco, CA, USA), on a series of clinical microbiology multiple-choice questions from Wu’s, *Self Assessment Q&A in Clinical Laboratory Science* (3). The topics of interest highlighted in the question set were bacteriology, virology, mycology, serology, and molecular diagnostics. We then assessed whether the addition of a clinical reference text, the *Manual of Clinical Microbiology* (4)*,* could improve this performance when combined with retrieval-augmented generation (5) (RAG), which works by extracting the most relevant chunks of text in the reference and appending it to the beginning of the user-supplied prompt. Publicly available interfaces to these models implement RAG when a document is supplied prior to prompting. We utilized these interfaces to perform the experiments in this work. See the supplemental methods for more details.

**Fig 1:**
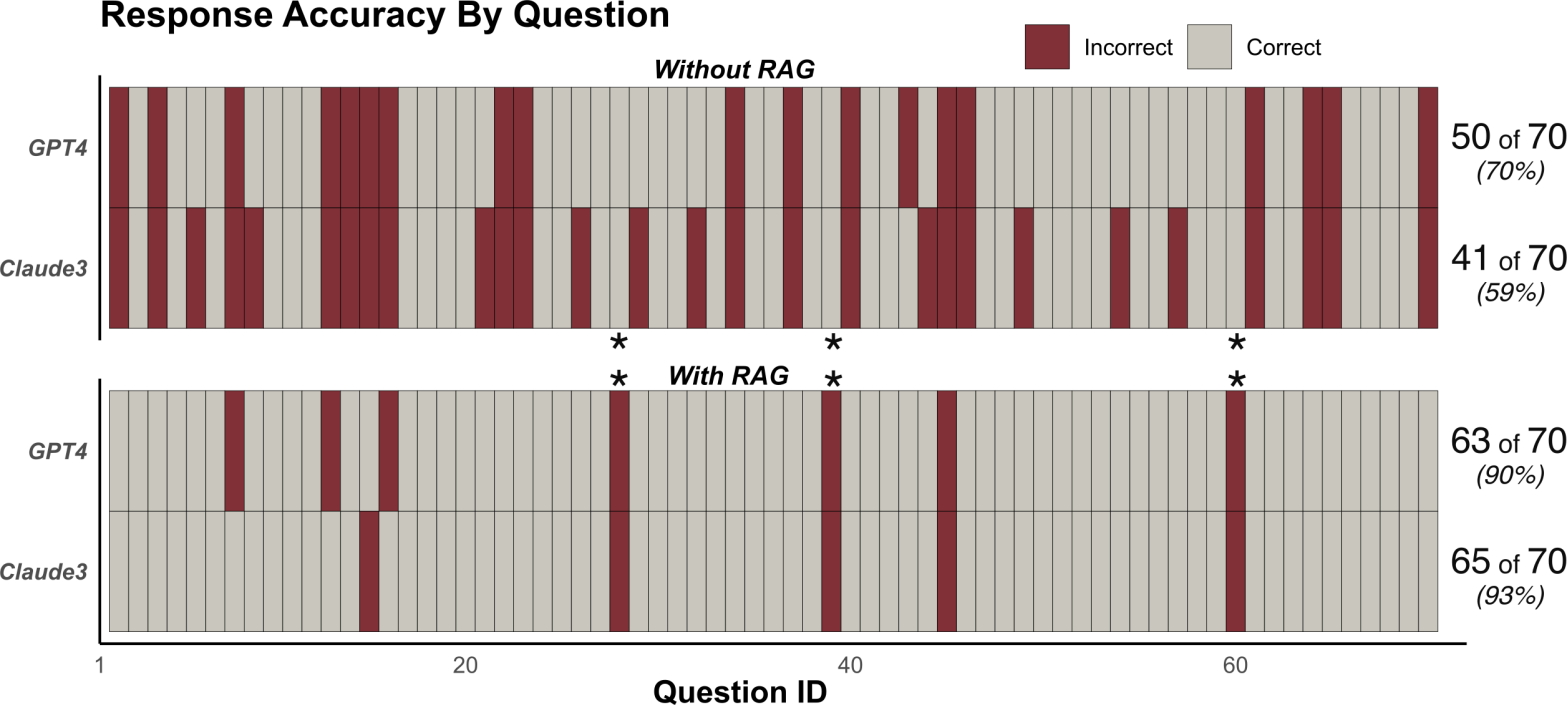
Response accuracy for two large language models with and without retrieval-augmented generation (RAG) for 70 boards style multiple-choice questions.

Without RAG, GPT-4 correctly answered 50 (71.4%) of 70 questions, whereas Claude3 Sonnet correctly answered 41 (58.6%). No significant difference between the models was observed (*P* = 0.3). The incorporation of a RAG step increased the accuracy to 90% for GPT-4 and 92.9% for Claude3 Sonnet, both significantly different from the off-the-shelf performances (*P* < 0.001). Figure 1 displays the question-wise accuracy for each model and framework.

Subjectively, the most notable generalization we observed from off-the-shelf responses was the propensity for hallucination. Incorrect justifications were provided with confidence indistinguishable from their correct counterparts, with factual errors often pertaining to biochemical or growth characteristics that would require significant expertise to detect (e.g., *Coxiella burnetii* testing positive for urease). These confident, but incorrect, responses pose a high risk for any potential clinical application.

Three questions answered correctly in the off-the-shelf experiments were answered incorrectly by both models in the RAG experiments. Question 28 covered an IgM-only Toxoplasma serology, which the off-the-shelf models appropriately interpreted as falsely positive. The RAG experiments extracted pertinent text that mentioned the propensity for IgM to turn positive before IgG but failed to recognize the relatively early emergence of IgG in *Toxoplasma* infection. Question 39, regarding *Helicobacter pylori* testing methods, was perhaps appropriately justified by the RAG experiments, in that antibody testing is thought to be more sensitive than antigen-based testing. Question 60, regarding nucleic acid amplification testing (NAAT) in cerebrospinal fluid, can be described as a semantic misunderstanding. The question asks which sources are *commonly* tested for by NAAT, which the RAG approaches responded *all of the above* while referencing appropriate sections for the reference text. Although NAAT assays do exist for all answer choices, they are not commonly used clinically for spirochetes or *Cryptococcus*.

These findings highlight that off-the-shelf LLM applications are unlikely to be ready for clinical use within clinical microbiology. However, through the addition of a RAG step to provide relevant and trustworthy clinical context, these models generate responses that are more likely to be factually accurate. This work is the first (to our knowledge) explicit evaluation of multiple LLM applications with and without RAG in clinical microbiology but comes with limitations. First, it is possible that despite copyrights, the evaluation material used in this work was present in the training corpus for the models, causing the off-the-shelf performance to be artificially overestimated. Second, although multiple-choice questions are a practical tool for evaluating baseline “knowledge,” the extent to which this observation can be extended to more clinically relevant applications, such as microbe identification, biochemical interpretation, susceptibility testing, and antimicrobial selection/stewardship is an opportunity for future exploration. Additionally, the text-based evaluation we present limits the potential use cases, and the extension of this work to image-based or multi-modal applications would present a valuable opportunity for automation or decision support.

Altogether, we believe that these results highlight the value of clinical context and subject-matter expertise in problems of clinical microbiology. We recommend approaching any LLM application without access to such context or expertise with extreme caution and encourage further exploration of techniques to overcome the current limitations of this potentially valuable technology.

## SUPPLEMENTAL MATERIAL

Supplemental methods jcm.01624-24-s0001.docx A brief overview of how these experiments were performed using the publicly available LLM RAG interfaces.

Table S1 jcm.01624-24-s0002.xlsx Question and answer brief summaries.

ASM does not own the copyrights to Supplemental Material that may be linked to, or accessed through, an article. The authors have granted ASM a non-exclusive, world-wide license to publish the Supplemental Material files. Please contact the corresponding author directly for reuse.
