# Supplementary material for: Retrieval-augmented generation salvages poor performance from large language models in answering microbiology-specific multiple-choice questions
Source: J Clin Microbiol. 2025 Feb 11;63(3):e01624-24. doi: 10.1128/jcm.01624-24 (PMC11898693; doi:10.1128/jcm.01624-24)

Supplementary Methods

This document outlines the methods for the Letter to the Editor, “Retrieval-Augmented Generation Salvages Poor Performance From Large Language Models in Answering Microbiology-Specific Multiple Choice Questions”, in which we sought to evaluate the performance of publicly available large language models on clinical microbiology multiple-choice questions. The process described below was performed in April 2024, using upgraded subscriptions to each service. Interface details, capabilities, and access may have changed since this writing.

## 1. Aggregating the Reference Text

First, the content chapters from the Manual of Clinical Microbiology, 11^th^ edition, were downloaded and combined. Due to upload size restrictions from the interfaces, four PDFs were generated; sections 1 and 2, sections 3 and 4, sections 5 and 6, and sections 7 through 9. These will be used as our reference material for the experiments that utilize the retrieval-augmented generation capabilities within the LLM interfaces.

## 2. Building the GPT

To streamline the process of repetitive question asking and answering, a custom GPT was built within the OpenAI interface. To do so, navigate to <https://chatgpt.com/gpts> and click “+ Create” in the top right corner, then “Configure” in the top panel. This will open the page shown below. Fill in the instructions with the desired pre-prompting information. The instructions for our letter are shown. Of note, we did not utilize the “Web Search” functionality, though its impact on speed and performance is worthy of future exploration.


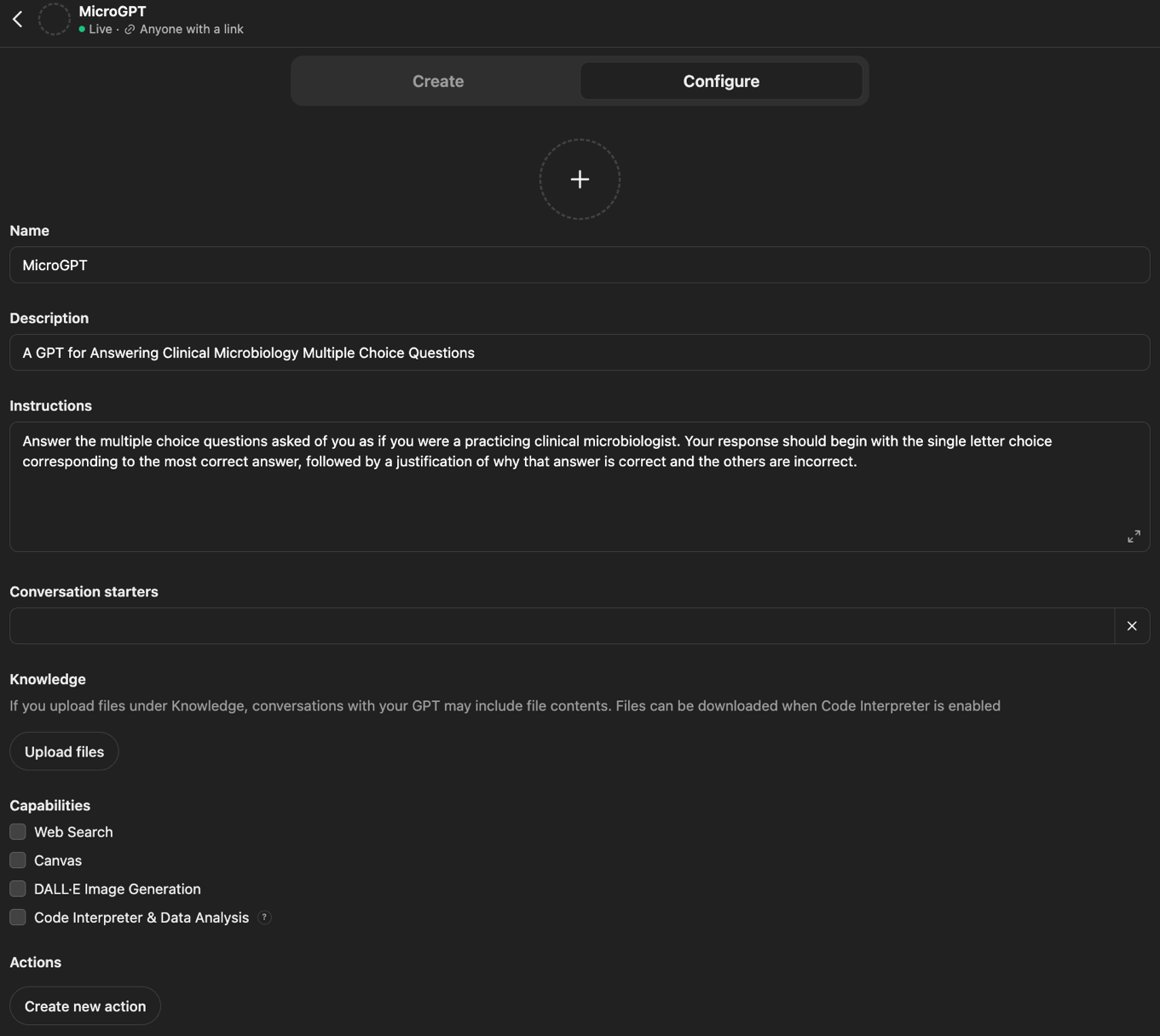


## 3. Prompt the GPT

Next, for each question in your question set, copy and paste the text into the prompt window on the right side of the screen, as shown below. Mark down the letter choice and justification from the GPT for each question. **Then, refresh the page prior to the next question.** This removes the previous question and answer from the GPT’s context window, allowing each question and answer to be independent observations from each other. Of note, at the time of writing, Claude3 did not have a mechanism to save a GPT with pre-instructions, so each question required pre-instructions to be uploaded prior to prompting, after starting a new session for each response.


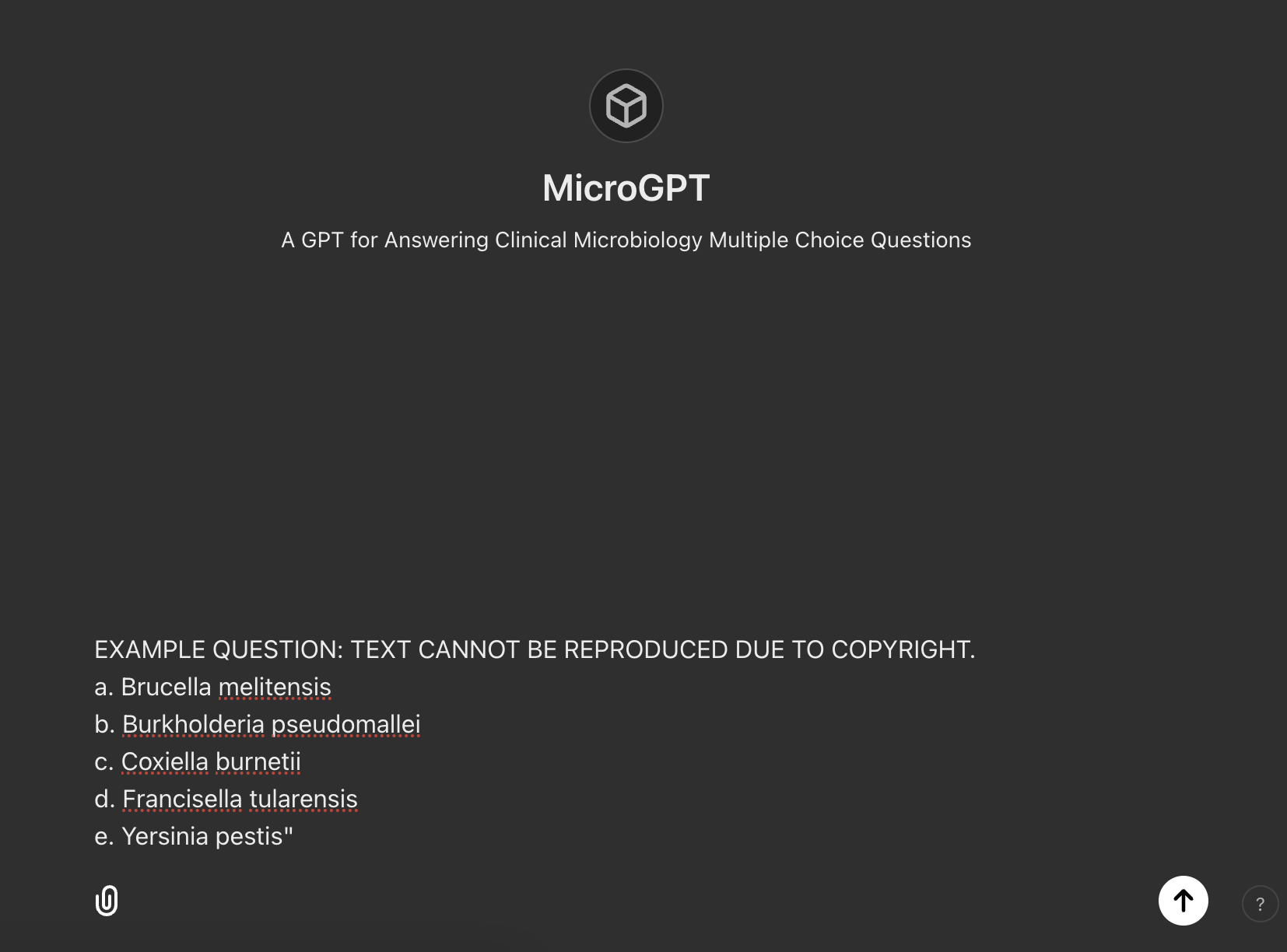


## 4. Incorporating RAG and the Reference Text

Next, to utilize the built-in RAG functionality, return to the “Configure” page of the GPT. Then, under the “Knowledge” sub-section, click “Upload Files” and upload the relevant reference texts you wish to use, shown below. Save the GPT, then repeat the prompting steps described in Step 3. Again, of note, at the time of writing, Claude3 did not have a mechanism to save a GPT with pre-instructions, so each question required pre-instructions and reference texts to be uploaded prior to prompting, after starting a new session for each response.


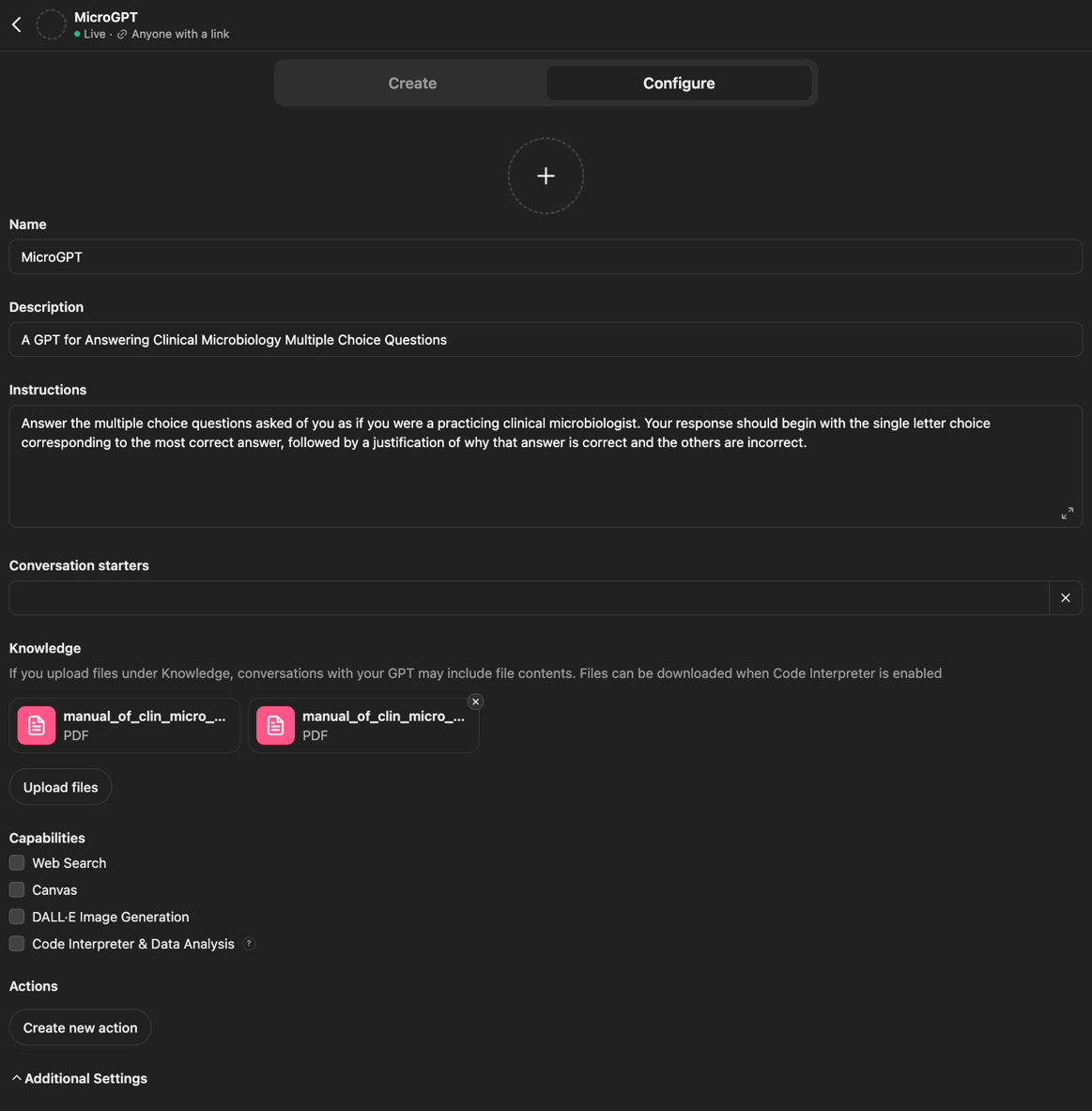

Supplement: Supplemental methods — A brief overview of how these experiments were performed using the publicly available LLM RAG interfaces. [file jcm.01624-24-s0001.docx]
